# Detection of D2-40 monoclonal antibody-labeled lymphatic vessel invasion in esophageal squamous cell carcinoma and its clinicopathologic significance

**DOI:** 10.7497/j.issn.2095-3941.2013.02.003

**Published:** 2013-06

**Authors:** Bing Bai, Wei Ma, Kai Wang, Sita Ha, Jian-Bo Wang, Bing-Xu Tan, Na-Na Wang, Sheng-Si Yang, Yi-Bin Jia, Yu-Feng Cheng

**Affiliations:** 1 Department of Radiation Oncology, Qilu Hospital of Shandong University, Jinan 250012, China;; 2 Department of Oncology, Yiyuan Chinese Medicine Hospital, Zibo 256100, China;; 3 Department of Oncology, Wendeng Central Hospital, Weihai 264400, China;; 4 Department of Cell Pathology, Graduate School of Medical Sciences, Kumamoto University, Kumamoto 8600862, Japan

**Keywords:** Esophageal squamous cell carcinoma, lymphatic vessel invasion, D2-40, lymph node metastasis, prognosis

## Abstract

**Objective:**

This study aims to investigate the clinicopathologic significance of lymphatic vessel invasion (LVI) labeled by D2-40 monoclonal antibody in esophageal squamous cell carcinoma (ESCC).

**Methods:**

Immunohistochemical assay was used to detect the expression of D2-40 and LVI in 107 ESCC patients. Then, the correlation between the clinicopathologic feature and the overall survival time of the patients was analyzed.

**Results:**

The lymph node metastasis rates were 70% and 21% in the LVI-positive and LVI-negative groups, respectively. The nodal metastasis rate was higher in the LVI-positive group than in the LVI-negative group. Multivariate regression analysis showed that LVI was related to nodal metastasis (*P*<0.001). The median survival time of the patients was 26 and 43 months in the LVI-positive and LVI-negative groups, respectively. Although univariate regression analysis showed significant difference between the two groups (*P*=0.014), multivariate regression analysis revealed that LVI was not an independent prognostic factor for overall survival in the ESCC patients (*P*=0.062). Lymphatic node metastasis (*P*=0.031), clinical stage (*P*=0.019), and residual tumor (*P*=0.026) were the independent prognostic factors.

**Conclusion:**

LVI labeled by D2-40 monoclonal antibody is a risk factor predictive of lymph node metastasis in ESCC patients.

## Introduction

Lymph node metastasis is an important basis for esophageal squamous cell carcinoma (ESCC) patients to prognose and choose a post-operative rehabilitation program^1^. Although tumor has been completely resected and lymph nodes have been widely cleared, local or general recurrence is still usual^2^. The survival rate in a 5-year period is only 15% to 39%^3^. In tongue squamous cell carcinoma and gastric carcinoma, LVI is closely related to lymph node metastasis^4^^,^^5^. Some reports also revealed that LVI is an independent risk factor for the prognosis of breast cancer and colon cancer^6^^,^^7^. Podoplanin is a type of mucous transmembrane protein expressed in lymphatic vessel endothelial cells^8^. The monoclonal antibody D2-40 can detect podoplanin^9^. In this study, D2-40 was used as a label to detect tumor cell invasion in lymphatic vessels of esophageal squamous carcinoma tissues, and the relationship among D2-40, clinicopathologic factors, and overall survival period was investigated.

## Materials and methods

### Specimens

A total of 107 ESCC specimens in paraffin blocks were stored from January 2007 to December 2007 in Qilu Hospital of Shandong University. Patients that were not treated chemically or radially were included in this study. After the operation, the patients were observed based on pathologic histology, with a follow-up period of 4 to 50 months (median: 40 months). The age of the patients ranged from 42 to 77 years old (median: 59 years old). The length of lesion ranged from 3 to 85 mm (average: 38.7 mm). Among the 107 patients, 14 had a lesion depth of T_1_, 23 had T_2_, 62 had T_3_, and 8 had T_4_. Also among the 107 patients, 29 had highly differentiated cancer, 53 from moderately differentiated cancer, and 25 had lowly differentiated cancer. The clinical staging is subjected to the tumor-node-metastasis (TNM) staging standard (version 6) by UICC in 2002. Among the 107 patients, 17 were in stage I, 53 in stage II, and 37 in stage III. After the cancers of the patients were resected, no cancer cells were observed in resection edge by microscopy in 102 patients, cancer cells were observed in 4 patients, and cancer tissues were observed by the naked eye in 1 patient. After operation, further standard therapy was adopted. For other information, see **Table 1**. This study was approved by the Ethics Committee of Qilu Hospital of Shandong University. Written informed consent was obtained from all patients.

**Table 1:** Relationship between LVI expression and clinicopathologic features of ESCC

Characteristics	*n* (%)	LVI		*χ*^2^	*P*
		Negative (%)	Positive (%)		
Gender	107	75 (70)	32 (30)	0.681	0.409
Male	85 (80)	58 (54)	27 (25)		
Female	22 (20)	17 (16)	5 (5)		
Age (years)				0.683	0.408
≤60	57 (53)	38 (36)	19 (18)		
>60	50 (47)	37 (34)	13 (12)		
Location				0.721	0.697
Upper	11 (10)	7 (7)	4 (4)		
Midthoracic	60 (56)	44 (41)	16 (15)		
Lower	36 (34)	24 (22)	12 (11)		
Length (mm)				4.729	0.030
≤36	54 (51)	43 (40)	11 (10)		
>36	53 (49)	32 (30)	21 (20)		
pT				5.057	0.025
T_1_ + T_2_	37 (35)	31 (30)	6 (5)		
T_3_ + T_4_	70 (65)	44 (41)	26 (24)		
Differentiation				0.068	0.794
G_1_ + G_2_	82 (77)	58 (54)	24 (22)		
G_3_	25 (23)	17 (16)	8 (8)		
TNM stage				23.564	<0.001
I + II	70 (65)	60 (56)	10 (9)		
III	37 (35)	15 (14)	22 (21)		
pN				22.020	<0.001
N_0_	69 (64)	59 (55)	10 (9)		
N_1_	38 (36)	16 (15)	22 (21)		

Note: pT, pathological T stage; G_1_, well differentiated; G_2_, moderately differentiated; G_3_, poorly differentiated; pN, pathological N stage.

### Methods

The ESCC tissue was fixed in 10% formaldehyde, embedded in paraffin, serially sliced into 4 μm segments, and stained by immunohistochemical s-p assay. The immunohistochemistry reagent was obtained from DAKO America. Known positive cervical squamous cell carcinoma slices were used as positive control, and phosphate-buffered saline was used to replace primary antibody incubation as negative control. The slices were observed by a low-power lens (×100). The single endothelial cell or endothelial cell cluster in the tumor tissues stained into brown yellow or chocolate brown was used as D2-40-positive expression. In the D2-40-positive lymphatic vessel cavity, ESCC cells were found. Thus, we preliminarily speculated that LVI was positive. This speculation was verified under a high-power lens (×400).

### Statistical analysis

The software SPSS 19.0 was used for statistical analysis of data. The Chi-square test was used to compare the relationship between esophageal squamous carcinoma LVI and clinicopathologic factors. In comparing the relationship between lymph node metastasis and clinicopathologic factors, Chi-square test or Fisher’s exact probability was adopted for univariate analysis, whereas binary logistic regression was adopted for multivariate analysis. The Kaplan-Meier method was adopted in analyzing the correlation between each clinicopathologic factor and prognosis of ESCC. Cox proportion risk regression model was used for multivariate analysis of lesion depth, lymph node metastasis, clinical staging, LVI, tumor residual, and so on. Statistical significance was considered at *P*<0.05.

## Results

### Characteristics of D2-40-positive lymphatic vessel and LVI in ESCC

**Figure 1:**
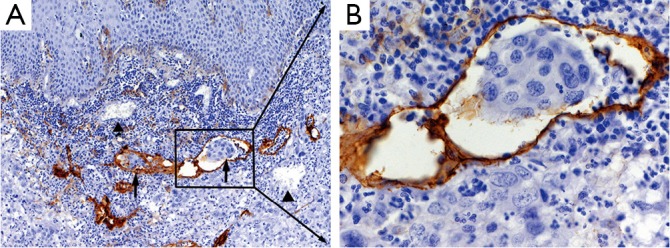
D2-40 expression in esophageal squamous cell carcinoma. A, ↑, Lymphatic vessel invasion (LVI) labeled by D2-40 monoclonal antibody; ▲, Endothelial cells in the blood vessels did not stain D2-40 (magnification, ×100); B, High power field (magnification, ×400).

D2-40-positive lymphatic vessels were visible in and near ESCC. The lymphatic vessels were tubular or cord-like, with thin duct wall, absence of erythrocytes in the cavity, and unstained adjacent blood vessels (**Figure 1A**). One, several, or a group of cancer cells were observed in some D2-40-positive lymphatic vessels (**Figure 1B**). LVI-positive expression was found in 32 patients, with a positive rate of 30%.

### Relationship between D2-40-positive LVI and clinicopathologic factors

No significant differences in LVI were found among the ESCC patients of various ages, gender, pathological locations, and differentiation levels. However, LVI was found in close relation to lesion length (*P*=0.030), depth (*P*=0.025), clinical stage (*P*<0.001), and lymph node metastasis (*P*<0.001) (**Table 1**).

### Relationship between D2-40-positive LVI and lymph node metastasis

The lymph node metastasis rates were 70% and 21% in the LVI-positive and LVI-negative groups, respectively. Multivariate analysis results showed a statistically significant difference between the two groups (*P*<0.001). However, in the univariate analysis of the relationship between other clinicopathologic factors and lymph node metastasis, the lymph node metastasis rates of the T_3_ and T_4_ groups were obliviously higher than those of the T_1_ and T_2_ groups (*P*=0.029). The lymph node metastasis rate of the low differentiated group was higher than that of the highly differentiated and moderately differentiated groups (*P*=0.049). However, the results of multivariate analysis revealed that lesion depth, length, and differentiation level were not independent risk factors for lymph node metastasis (**Table 2**).

**Table 2:** Relationship between lymph node metastasis and clinicopathologic features of ESCC

Variables	Univariate			Multivariate		
	*χ*^2^	*P*		HR	95% CI	*P*
Gender	0.337	0.562		–	–	–
Age	0.178	0.673		–	–	–
Location	0.065	0.968		–	–	–
Length	0.840	0.359		–	–	–
pT	7.051	0.008		1.994	0.987–4.027	0.054
Differentiation	0.330	0.565		–	–	–
TNM stage	12.701	<0.001		2.067	1.129–3.785	0.019
pN	8.959	0.003		1.893	1.060–3.381	0.031
Residual tumor	10.035	0.002		3.042	1.146–8.079	0.026

Note: pT, pathological T stage; pN, pathological N stage.

### Relationship between D2-40-positive LVI and prognosis of ESCC patients

The median survival time of the LVI-negative and LVI-positive groups was 43 and 26 months, respectively. The median survival time of the negative group was significantly longer than that of the positive group, as indicated by univariate analysis (*P*=0.014). However, in the multivariate analysis of lesion depth, length, lymph node metastasis, clinical stage, tumor residual, and so on, LVI was not an independent risk factor for prognosis (*P*=0.062). The lymph node metastasis (*P*=0.031), clinical stage (*P*=0.019), and tumor residual (*P*=0.026) were in close relation to prognosis (**Table 3**).

**Table 3:** Relationship between overall survival and clinicopathologic features of esophageal ESCC

Variables	Univariate			Multivariate		
	*χ*^2^	*P*		OR	95% CI	*P*
Gender	0.165	0.684		–	–	–
Age	0.094	0.759		–	–	–
Location	1.889	0.169		–	–	–
Length	0.009	0.925		–	–	–
PT	4.766	0.029		2.548	0.881-7.367	0.084
Differentiation	3.871	0.049		2.955	1.000-8.729	0.050
LVI	22.02	<0.001		8.933	3.193-24.988	<0.001

Note: pT, pathological T stage.

## Discussion

LVI is conventionally detected by observing hematoxylin and eosin (H&E) stained specimens with an optical microscope. However, distinguishing LVI in H&E staining is difficult because of the following reasons: (1) lymphatic vessel wall is thin and is thus easily compressed and usually neglected in microscopy; (2) distinguishing small cancer nests from the lymphatic vessel cavity filled with clustered tumor cells is difficult; (3) during fixing tumor tissues, isolated tumor cell clusters are generated by tissue compression, causing the tumor cell clusters to be usually confused with cancer embolus in lymphatic vessels^10^. Podoplanin protein is an antigen separated from the testis of a fetus^11^^,^^12^ and is specifically expressed in lymphatic vessel endothelial cells. The monoclonal antibody D2-40 labeling lymphatic vessels in this study can detect podoplanin protein. D2-40 is featured by high specificity and sensibility for detecting lymphatic vessels^9^.

No uniform quantitative standard was used for judging cancer cell invasion in D2-40-positive lymphatics, making the judgment results easily affected by human factors. In this study, two pathologists observed slices jointly. If both of them judged LVI to be positive, then it was deemed positive. In this study, D2-40 was used to detect LVI. A positive rate of 30% was obtained. In previous studies, the results of D2-40 detection yielded an LVI-positive rate of 30% to 79%^10^^,^^13^. The difference was caused mainly by the proportion of patients positive for lymph node metastasis. In the 107 ESCC patients in this study, the positive rate of lymphatic metastasis was 32% (38 cases). This result was verified by univariate and multivariate analyses. LVI was found in close relation to lymph node metastasis of ESCC. This result is consistent with the results reported by other studies^10^^,^^13^^,^^14^. In addition, LVI was found in close relation to lesion length, depth, and clinical stage. This result reflects tumor invasion and indicates that most advanced cancers are a type of disease that is not limited to tumor itself but a general disease.

Multivariate analysis of the relationship between D2-40-labeled LVI and prognosis showed a close relation (*P*=0.014). However, in multivariate analysis, LVI was not an independent risk factor for the prognosis of ESCC patients (*P*=0.062). Lymph node metastasis (*P*=0.031), clinical stage (*P*=0.019), and tumor residual (*P*=0.026) were found in close relation to the prognosis of ESCC. These results indicate that lymph node metastasis is an intermediate step for correlating LVI with the prognosis of ESCC. LVI occurs before lymph node metastasis, and can be used as a predicative factor for lymph node metastasis. Some studies reported that D2-40 labeled LVI is closely related to the clinical prognosis of ESCC^10^^,^^13^. However, some studies have drawn an opposite conclusion^14^^,^^15^. This difference may be caused by follow-up time, post-operative therapy, and so on. The relationship between D2-40-labeled LVI and prognosis of ESCC need to be further studied with more clinical data.

ESCC patients with negative lymph node metastasis suffered from a recurrence rate of 20% to 45% after operation. This result can be attributed to the fact that lymph node micro-metastasis occurred during the operation. Detecting micro-metastasis clinically is not practical. Identification of LVI provides a very good way for predicting lymph node metastasis.

In conclusion, using D2-40 to detect LVI is of significance in predicting lymph node metastasis of ESCC. D2-40 can be used for clinicalpathologic detection and for determining ESCC stage and rehabilitation program.
